# Exploitation Perspective Index as a Support of the Management of the Transformer Fleet

**DOI:** 10.3390/s23218681

**Published:** 2023-10-24

**Authors:** Michał Kunicki, Sebastian Borucki, Jan Fulneček

**Affiliations:** 1 Department of Electrical Power Engineering and Renewable Energy, Opole University of Technology, 45-758 Opole, Poland; weai@po.edu.pl; 2 Department of Electrical Power Engineering, VSB Technical University of Ostrava, 708 00 Ostrava, Czech Republic; jan.fulnecek@vsb.cz

**Keywords:** power transformer, high voltage, measurements, condition assessment, fault diagnostics

## Abstract

This paper presents an alternative approach to the Transformer Assessment Index (TAI) by proposing a relatively simple rating method called the Exploitation Perspective Index (EPI). The method provides two numerical indicators: the first reflects the overall technical condition of the particular unit, and the second shows the condition of the unit in the context of the entire fleet. The objective of the EPI method is to support the decision-making process regarding the technical condition assessment of each of the transformers in the target population, considering not only technical but also economic aspects of transformer maintenance. Application of the method is described step by step, including input data, parametrization of the weights, and interpretation of the output results it provides. The proposed method is evaluated by two representative use cases and compared with two other methods. As a result, EPI confirms its applicability, and it has already been successfully implemented by the electric power industry. EPI can be potentially freely adopted for any transformer fleet, as well as for the specific situation of the utility, by adjusting the relevant parameters.

## 1. Introduction

One of the priorities for utilities is to provide uninterrupted power supply to end users. The reliability of the electric power asset mainly depends on adequate maintenance and diagnostics [1,2,3,4]. Power transformers are no doubt one of the key elements of the entire power system, so their technical condition, especially faults, may directly affect the reliability of the system. Condition assessment of transformers is based on various measurement data, usually aimed at assessment of the insulation system and mechanical condition [5,6,7,8], as well as the influence of physical factors like temperature [9,10,11], load, and even weather [12]. Knowing the technical condition of a particular unit is only the first step in the maintenance and risk management chain regarding the entire fleet. Adequate management of the transformer fleet requires additional decision support tools, which indicate the absolute technical condition of a given unit in the context of the entire fleet. Such tools are called the Transformer Assessment Index (TAI) and may be implemented in various ways, briefly discussed below [13].

In [14], the authors considered the unavailability of the data as one of the limitations of TAI. In their opinion, such a situation may result in inadequate condition assessment of a transformer due to limited data calculation. To solve this problem, the authors proposed the application of parameter prioritization results to estimate the certainty level caused by the data availability of a TAI. To demonstrate the proposed, method five scenarios were used to calculate the certainty level. Validation of the proposed approach in the context of its influence on TAI was performed on a 150 kV power transformer with six points of measurement data. This problem was also raised by the same authors in [15], where a comparison of seven models to substitute unavailable data on paper insulation condition was presented. The health index of 200 transformers with complete data was calculated, and compared to the alternative models. The analysis showed that the optimized models were based on multiple linear regression and an adaptive neuro-fuzzy inference system. The problem of adjusting the weighting factor of TAI was raised in [16]. According to the authors, one of the possible solutions to this problem is the involvement of many experts, but usually such an approach leads to complexity in aggregating the results. As a result, a novel method to implement the consensus for multiple experts in transformer assessment index weighting factor de-termination based on the analytic hierarchy process was proposed. Aman et al. in [17] proposed a novel method for assessing the overall health condition of the transformer. A combination of the health index and the criticality index was used to prioritize the strategic decisions on the transformer. The proposed method divided the population into four groups, taking into account diagnostic indicators and the critical operations of individual transformers. Consideration of the transformer’s apparent and actual age and their potential influence on the assessment of its aging condition was presented in [18]. The authors calculated the health index for 130 units and used them to model the curve of the heath index decrease in the entire population. The apparent age and aging condition of the transformer were then estimated using the proposed curve. The apparent age problem also appeared in [19], where a novel method for assessing the health index of transformers, considering the apparent age strategy, was proposed. The method used four input factors for the evaluation process (DGA, oil quality, bushings conditions, and equipment degradation). Apparent age was included in the method mainly by hot spot temperature and load data. In [20], the authors investigated the improvement of the TAI by using fuzzy logic methods. The integrated fuzzy model was developed by combining the sub-models of fuzzy logic. The proposed solution yielded an improved assessment of the transformer insulation condition. According to the authors, this approach decreases the complexity of life estimation and health index evaluation of power transformers. Another modeling approach regarding TAI was proposed in [21]. Zeinoddini-Meymand et al. analyzed the application of linear and nonlinear models to evaluate TAI. The authors compared two nonlinear models (artificial neural network and adaptive neuro-fuzzy inference system) with a multiple linear regression linear statistical model. Furthermore, they proposed an extended data set to optimize the TAI calculation, including 15 parameters (i.e., DGA and various oil properties). This study showed that an adaptive neuro-fuzzy inference system-based model provided the best results. A newly developed TAI was also proposed in [22]. A hierarchical health index model was established based on statistical product and service solutions. The proposed method takes into account the perspectives of transformer thermal, electrical, mechanical, and load. An interesting aspect was raised by Benhmed et al. in [23], where the authors investigated feature selection and classification techniques to reduce the complexities of TAI. Several filters and wrapper-based feature selection methods were investigated. The performance of the proposed approach is validated by evaluations of selected classification models. According to the authors, their method reduced the optimum number of features by separating only the most influential ones when calculating TAI. Furthermore, results showed that water content, acidity, breakdown voltage, and furans were the most influential testing parameters in calculating TAI. A novel health index, risk, and remaining lifetime estimation method for power transformers is discussed in [24]. The method was based on a combination of three selected models: a winding degradation physical model, a health index model based on condition monitoring data combined with expert judgment, and a statistics-based end-of-life model. Data from real-life transformers were used to validate the method. According to the authors, this method allows for the identification of transformers in poor condition and the follow-up and prioritization of transformers for maintenance and replacement.

Some of the current studies on TAI, apart from the assessment of the technical condition, also take into account economic aspects. In [25], the authors proposed an innovative approach to the maintenance decision-making model, considering reliability and cost-effectiveness. This approach was based on a particle swarm optimization method, which was used to optimize the proposed model and select the best maintenance strategy. The performance of the proposed method was verified in two cases, which confirmed a significant improvement in the maintenance strategy with this method. In [26], the authors used a health index to assess the maintenance cost of transformers. This method is based on the Markov model, which is used to predict the future state of the transformer. Future-state distribution probabilities were used to estimate the maintenance cost of the selected unit, according to the proposed maintenance policy model.

Further discussion and comparison of various contemporary approaches to TAI can be found in [13,27,28].

Thus, this paper proposes an alternative technical condition index method for power transformers called Exploitation Perspective Index (EPI). The objective of this method is to provide a simple, universal, and personalized tool to provide a brief view of the entire fleet as well as a particular transformer. The most relevant novelty regarding this method is that it takes into account both the technical condition of the assessed transformer and the economic aspect of the required maintenance. Furthermore, EPI provides two rating scales in one rating procedure: absolute and relative. The absolute scale corresponds with the current technical condition of the unit and its potential future exploitation perspective, while the relative scale reflects the overall technical condition of the particular unit in the context of the entire fleet. Finally, potentially, EPI can be freely adopted for any transformer fleet as well as for the specific situation of the utility by adjusting the relevant parameters.

## 2. Proposed EPI Method

### 2.1. Requirements for the Proposed Method

This section raises some key assumptions the proposed EPI method should meet regarding its functionality and range of applications. Most of the assumptions for EPI are derived from typical requirements that similar methods should comply with. Most of these requirements are grounded in applicable industry standards and common good practices in the field of technical condition assessment of power transformers. However, it must be emphasized that the EPI method is designed for a specific target transformer fleet, and as a result, some of the requirements and proposed assumptions may apply strictly to this fleet.

- The objective of the EPI method is to support the decision-making process regarding the technical condition assessment of each transformer in the target population;
- The method should yield a simple rating to show the overall technical condition of the particular unit in the context of the entire fleet;
- The method should use the conclusions from periodical routine test results (instead of raw measurement data) of the transformers typically performed in the fleet the EPI is designed for;
- EPI should not analyze any of the raw measurement data, while it should rather use an expert diagnosis (defects and other malfunctions detected on the grounds of the routine tests) for further analysis;
- EPI should be a numerical value that corresponds with the current technical condition of the unit and its potential future exploitation perspective (absolute rating scale);
- EPI should focus not only on technical but also economic aspects of transformer maintenance;
- EPI should also reflect the overall technical condition and future exploitation perspective of the particular unit in the context of the entire fleet (relative rating scale).

### 2.2. Description of the Proposed Method

#### 2.2.1. Input Data Initial Preparation 

One of the crucial steps prior to the implementation of EPI is the initial preparation of the input data. All of the inputs should be taken from the periodic transformer test reports. It should be emphasized that EPI does not analyze any raw measurement data, so it does not need any new technical rating criteria or testing procedures to be defined. It is assumed that experts or decision support systems identify potential defects and draw auxiliary conclusions (based on the analysis of raw measurement data). Therefore, to prepare input data for EPI, appropriate standardization of the transformer test report is required. To address this point, it is proposed to include a predefined checklist in the conclusion section of every report. The idea is not to influence any test procedures or diagnostic criteria, but only to present the final conclusions in a categorized and predefined way. According to this proposal, the supplementary task for the expert (or system) diagnosing the analyzed transformer (in the test report) would be to mark the relevant defects/conclusions in the attached checklist (simple binary criterion): mark it if applicable, and not to mark it if not applicable (based on the analysis of the measurement results). Definitions and interpretations of the proposed input parameters are provided in Table 1. This list contains most (or even all) of the typical defects that may be identified in the transformer. 

#### 2.2.2. Implementation of EPI

**Figure 1:**
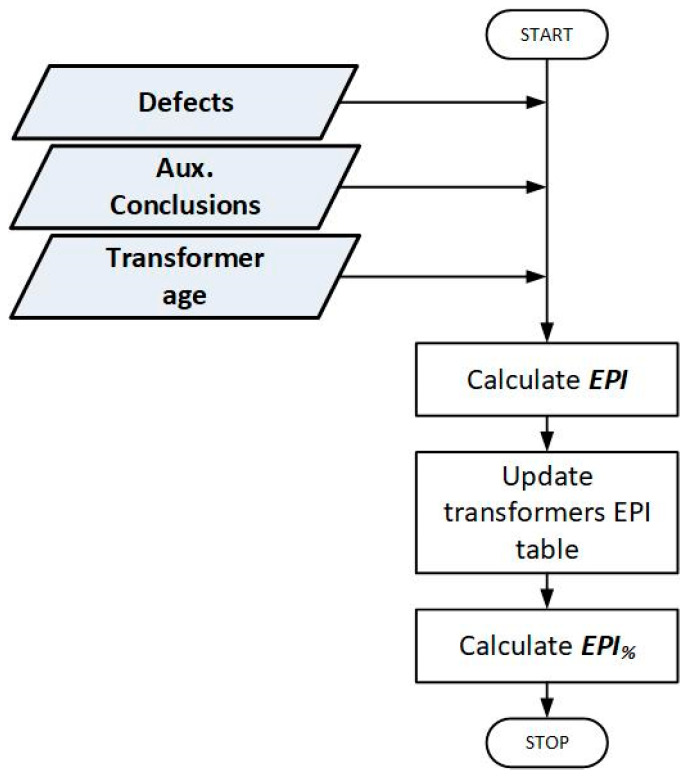
General flowchart of the EPI method.

EPI is a relatively simple, dimensionless rating method for transformers that considers not only their technical condition but also an economic aspect of the required maintenance. The proposed rating procedure is based on the results of actual periodic routine tests of the transformer. EPI uses 2 types of data as input parameters (Table 2): defects (DEF) and auxiliary conclusions (AC). Furthermore, it also considers the transformer age. As described in Section 2.2.1, EPI does not analyze any raw measurement data, instead using conclusions from the periodic transformer test report as input. It is quite different from other methods discussed in Section 1 and makes this method unique in this regard. The procedure for determining the EPI consists of summing up the weights assigned to all DEF and AC, respectively, indicated for a given unit (Table 2). It follows that theoretically, the minimum possible value of the EPI is equal to 0—in a situation where an assessed unit has no identified defects and failures, and thus no maintenance recommendation has been generated for it. Therefore, the maximum value of the EPI is theoretically limited by the sum of the weights of all DEF and AC (but it is a theoretical case rather than a real-life scenario). A general flowchart of the EPI method is presented in Figure 1.

A general formula to calculate the EPI for a selected transformer *tr* is presented in (1):

(1) $$EPI_{tr}=\sum_{i}D_{i},$$

where

(2) $$D_{i}=\left(d_{1}\cdot \frac{k_{i}\cdot m_{i}}{S_{tr}}+d_{2}\cdot n_{i}\cdot P_{tr}\right)\cdot 100,$$

is a single weight related to a specific *i*-th input parameter of the analyzed transformer, according to Table 1. Equation (2) consists of two fundamental parts related to the economical and technical aspects of the assessed unit, respectively. These formulas are empirically based on the experience of authors and industry experts; they also correspond to industry standards and recommendations in this regard [13].

First, the economical-related part uses *d*_1_, *k_i_*, *m_i_*, and *S_tr_* for calculation. *S_tr_* is an estimated value (price) of the new transformer meeting the same requirements as the assessed one (equivalent). Despite *S_tr_* having the same value for all input parameters (within the same assessment process), it may change over time, so it should be updated regularly. Estimated repair/replace/maintenance costs related to the *i*-th parameter are defined as *m_i_*. This parameter has different values for every input parameter, as they require different maintenance procedures (and consequently costs). Its value may also vary over time, so it should be updated if necessary. Both *S_tr_* and *m_i_* should be expressed in the same currency. *k_i_* can be defined as the economical weight of the *i*-th input parameter. Primarily, it should consider the age of the transformer (its depreciation), as usually some extended overhaul of the old units may not be rational from an economic point of view. Furthermore, *k_i_* can also consider some other aspects that may be important from an economic point of view, e.g., possible transport costs of the transformer, availability of specific components at a given moment, etc. The default value of *k_i_* for this EPI method is 1. In order to include the age of the transformer, one of the commonly proposed normalized aging (or loss of life, etc.) curves may be used [29,30,31], or it may be assigned on the basis of the experience and exploitation history regarding the particular utility. The *d*_1_ is an additional factor that depends on the general economic situation of the owner of the transformer. Its default value is 1, and if needed, it can adjust the relative importance of the economic aspect, depending on the specific market situation at a given time (the higher the *d*_1_, the greater the influence of the economic aspect on the final EPI value).

**Figure 2:**
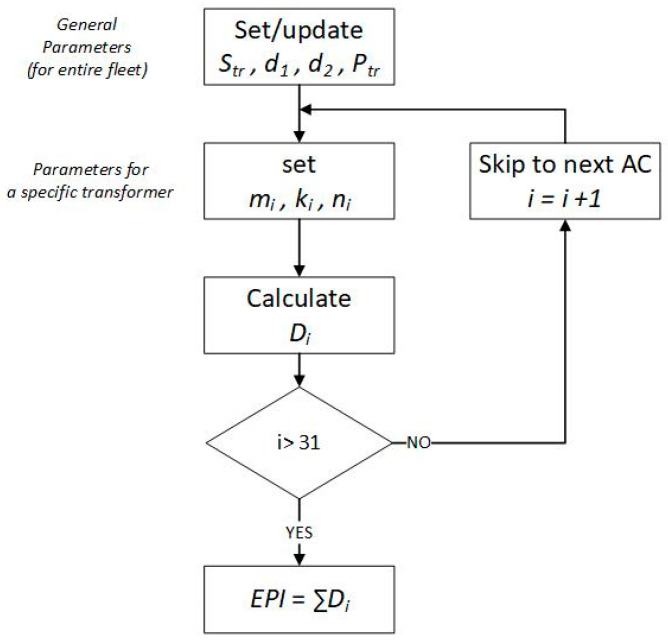
Flowchart of initialization of parameters for EPI calculation of the specific transformer.

The second part of (2) is related to the technical aspect of the analyzed transformer and uses *d*_2_, *n_i_*, and *P_tr_* for calculation. *P_tr_* represents the relative priority index of the assessed unit in the entire population. The default value of *P_tr_* is 1—lower priority represents values below 1, and higher priority represents values greater than 1. The higher the value of *P_tr_*, the greater the influence of the technical aspect on the final EPI rating. The importance of a specific input parameter from the transformer exploitation point of view is reflected in *n_i_*. Its value theoretically can vary from 0 to 1, where 0 means “problem irrelevant to the exploitation perspective” and 1 means “decommissioning of the unit/unable to operate and repair”. The *d*_2_ is an additional factor that depends on the general technical situation of the owner of the transformer. For example, it can reflect the current availability of certain resources (spare parts, spare units, repair crews, the possibility of stoppage, etc.). Its default value is 1, and if needed, it can adjust the relative importance of the technical aspect depending on the specific situation at a given time (the higher the *d*_2_, the greater the influence of the technical aspect on the final EPI value). Figure 2 illustrates the EPI calculation process for a given transformer.

Multiplication by 100 in (2) is not relevant from the EPI method point of view. However, in this case, it was included at the request of the utility this method was developed for to make the interpretation of the EPI somewhat similar to the “transformer wear percentage”. To illustrate the step-by-step calculation of *D*_i,_ moisture in the oil (*D*_18_) will be used below as an example, as it is one of the most common defects in oil transformers. Starting with the first part of (2), *d*_1_ is set to 1 (default value), as the general economic situation of the owner is normal. The estimated value of *m*_18_/*S*_tr_ is about 0.02, assuming that oil refining is recommended (if replacement of the oil is recommended, then it will be about 0.04), and the new unit value is approximately 1 mln € (typical 31 MVA unit). *k*_18_ is set to 0.5, as the economical relative aspect of this defect is rather minor (typical procedure of oil treatment is needed, which is commonly available and does not require any additional costs), and the age of the transformers is assumed as the mean of the fleet. So, the final value of the first part of (2) is now 0.01. For the second part of (2), *d*_2_ is set to 1 (default value), as the general technical situation of the owner of the transformer is normal. *n*_18_ is set to 0.05, as this is a typical defect that, if controlled and fixed, does not significantly affect the exploitation of the transformer. The priority of the transformer is set to the default value, *P*_tr_ = 1. So, the final value of the second part of (2) is now 0.05. Finally, adding two parts and multiplying by 100 gives the value of *D*_18_ = 6.

Table 2 presents a complete catalog of all possible input parameters for EPI and their proposed default values *D*_i_ assigned for the transformer population the EPI method was designed for. These values were calculated using (1) and (2), and specific values or parameters of (2) are mainly based on the experience of the authors, the suggestions and requirements of the owner of the fleet, and the specificity of the target user market. They also consider contemporary transformer exploitation guides, the expertise of the authors, and specialists from the utility in such a way as to reflect the importance of the indicated problems in the analyzed transformer and also take into account economic aspects, such as repair costs, carrying amounts, etc. [32,33]. As a result, proposed *D*_i_ values are not universal and should be adjusted to the specific fleet in which this system is to be used. However, the weights proposed in this study can be used as a starting point or reference in any case.

#### 2.2.3. Absolute Rating Scale

The proposed EPI method allows for two types of transformer evaluation: absolute and relative. The absolute scale is based on a typical three-point rating and uses strictly assigned boundaries for each rating (Table 3). After determining the value of the EPI, a given transformer unit is classified into one of three groups, divided in terms of its current technical condition and operational measures necessary to implement, aimed at ensuring its further trouble-free operation [33]. The first group includes transformers whose technical condition does not raise any major objections or requires only minor operational procedures that do not really affect the safety of their further operation. In practice, these are transformers for which no defects and faults have been shown, or only these that do not pose a direct threat to further trouble-free operation of a given transformer have been shown. The first group will include units for which the EPI parameter is less than 10—this group has been labeled *Does not require significant operational/investment procedures* and may be marked as green (in line with the usual practice in this regard [13]).

The second group is labeled *Required maintenance/investments*, and may be marked as yellow. It includes the transformers in relatively good but not perfect technical condition. It means that in order to ensure their continued reliable operation, it is recommended (sometimes even necessary) to implement in the near future some specific measures necessary for the safety of this unit. Failure to implement the procedures may, in the near future, result in an emergency shutdown of the transformer or its damage. The values of the EPI parameter that result in assigning a given transformer to this group range from 10 to 50.

The last, third group are classified units in the worst technical condition, indicating a relatively high probability of failure or requiring immediate key operational measures, without which their further operation may be at risk or even impossible. This group is labeled *Significant operational/investment measures required* and may be marked as red.

#### 2.2.4. Relative Rating Scale

Regardless of the absolute scale, EPI supports the relative evaluation scale of the assessed transformers in the context of the entire population (EPI_%_). The idea of the EPI_%_ is to indicate the technical condition of the assessed unit compared to the rest of the population. It requires gathering the EPI for all units in the population in one table. This table is used to calculate the percentile of the EPI for the entire population. As a result, the user obtains statistical information (EPI_%_), which shows what percentage of all units are in better and worse technical condition than the analyzed one. Such information complements the absolute EPI results, which may not be fully informed, especially regarding the perspective of the entire population. Moreover, the use of percentiles enables one to easily evaluate the conventional rating scale (absolute scale)—one only needs to select adequate percentile boundaries for each scale (i.e., in the case of a typical 5-grade rating, percentiles can be selected as follows: 0–20, 20–40, 40–60, 60–80, 80–100). In fact, this approach yields dynamic rating scales, as the boundaries are defined in a relative way (percentiles)—as a result, the absolute values of the boundaries will dynamically change as new data (new EPI) feeds the database. In practice, gathering the EPI of all transformers in the population may be very difficult (time consuming) or even impossible to achieve over a rational time horizon. Thus, alternatively, some representative sample of the population may be assigned with respect to the technical condition (and age) structure of the entire population (Figure 2).

## 3. Results and Discussion

This section shows the verification of the EPI method based on real-life scenarios in comparison with two other methods. The first method (HI1) was an industry standard transformer condition assessment method, which uses a table-based health index (scoring matrix). It means that diagnostic criteria are predefined and divided into several relevant categories and have the same values (boundaries) for the entire population. Each category has a 5-level rating scale (0–4 points), and the final rating is a simple sum of the ratings of all categories (Table 4). The higher the rating, the worse the technical condition of the transformer. In this study, seven categories were used for HI1: oil, solid insulation, windings, core, OLTC, bushings, and others (so the final HI1 value is between 0 and 28). Further details on the HI1 method can be found in [13].

The second method (HI2) employed an algorithm that used advanced oil diagnostics. In particular, it can be read as an improved standard method that uses three main diagnostic categories: basic diagnostics, active parts, and aging processes. Also, HI2 uses a number of predefined weights, which must be applied to different input parameters prior to the final rating calculation. Finally, the higher the rating, the worse the technical condition of the transformer. The final HI2 value is between 0 and 100%, which is divided into three states describing the technical condition of the diagnosed transformer (Table 5). More details on HI2 are presented in [34].

**Figure 3:**
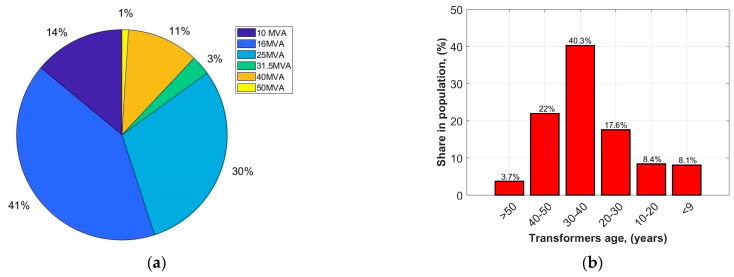
Rated power (**a**) and age structure (**b**) of the representative transformer population.

To illustrate the performance of this method, a specific transformer population was used—this population is a property of the utility the EPI method was designed for. The population consists of over 1500 units; their rated powers vary between 10 and 80 MVA; and their typical rated voltages are 115/16.5 kV (sometimes there are units with secondary rated voltages of 22 kV, 6 kV, or three-winding units). It was not practically possible to analyze the technical condition (to perform routine tests) of all of the units in the accepted time frames, so a representative sample was used instead. The selected sample statistically reflects the technical condition, age structure, and working conditions of the entire population and consists of 300 units (Figure 3).

**Figure 4:**
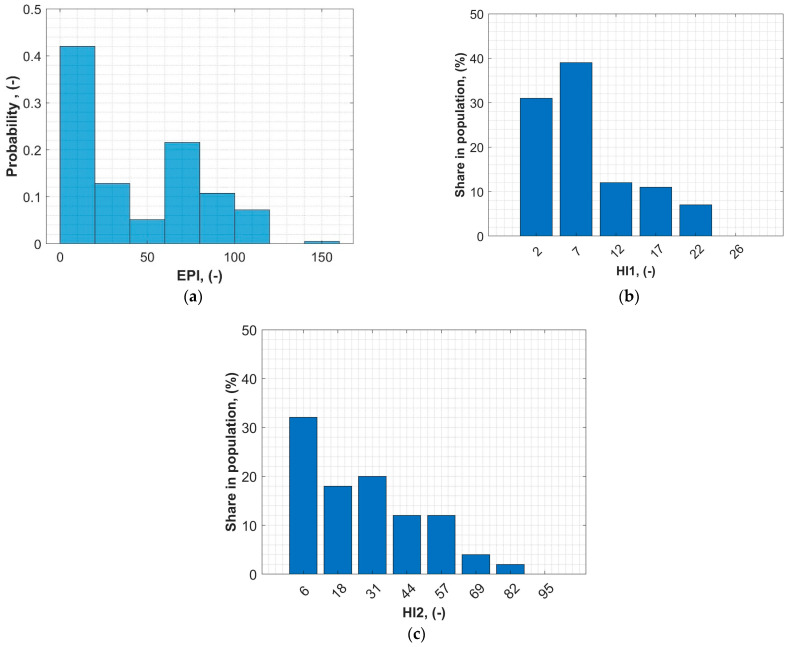
Histogram of technical condition assessment results in the analyzed population using different methods: (**a**) EPI, (**b**) HI1, and (**c**) HI2.

For these units, a complete routine test was performed, the results of which were used to evaluate the EPI method. Figure 4 shows the normalized histograms of EPI results compared with the HI1 and HI2 methods. According to EPI, it is noticeable that two groups are dominant: units in very good technical condition (EPI < 20, which covers over 40% of the population) and units in quite bad technical condition (EPI > 60, which covers another approx. 40% of the population). The remaining units are in moderate technical condition (20 < EPI < 60). According to the presented method, the percentile value for a given EPI read from Figure 3 is called EPI%. According to HI1, almost 70% of the population is in relatively good condition (HI1 > 10). It is a typical situation with this kind of method. As it uses several categories, poor conditions related to only one category may be masked by relatively good results in other categories, and the final assessment may not be relevant in such a case. Comparing HI2 and EPI, the results are quite similar. However, in the case of HI2, it is also characteristic that most of the units were assessed as in good or average condition (HI2 < 57). This is probably the result of HI2 not taking into account the economic aspects of the transformer’s technical condition and using only three diagnostic categories. On the other hand, HI1 and HI2, contrary to EPI, use raw measurement data to assess the transformer, which requires fixed diagnostic criteria. As a result, some specific malfunctions or defects may not be detected and included in the final assessment.

### 3.1. Use Case Tr1

This section describes an exemplary use case of the EPI method for the transformer in moderate conditions. In this case, the assessment was performed for a two-winding 40 MVA unit, 115/22 kV, which was manufactured in 2014 (Tr1). Table 6 presents an expert diagnosis and EPI results compared to HI1 and HI2 for Tr1. The diagnosis was based on the complete measurement data and visual inspection checklist issued after routine periodic tests. Expert diagnosis was used by the EPI to perform the assessment.

**Figure 5:**
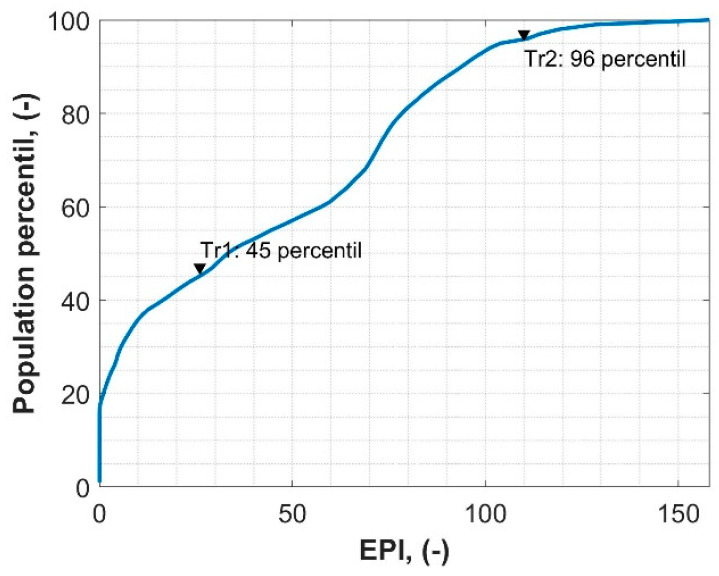
Percentile plot of EPI results in the analyzed population.

Tr1 was detected with two typical defects: moisture in the oil and in the solid insulation. These defects are quite common and usually do not imply any serious consequences, as long as they do not exceed specific thresholds. In this case, EPI is the sum of D_6_ and D_20_ according to Table 1, which is 26. According to the proposed absolute EPI rating scale (Table 2), Tr1 has a rating of two, and is classified as *Required maintenance/investments*. To know the value of EPI% (in the proposed relative rating scale), a percentile distribution of the entire population must be known. In this case, the value of EPI% for Tr1 is 45%, which means that 45% of units in this population are in better technical condition than Tr1, and 55% are in worse condition (Figure 5). Results for HI1 and HI2 are quite similar to those for EPI. As the analyzed case is rather typical, both methods show that this transformer is in moderate condition and that some maintenance is needed in the near future.

### 3.2. Use Case Tr2

This section presents the second exemplary use case of the EPI method for a transformer in poor technical condition. In this case, the EPI was performed for a three-winding 40 MVA unit, 115/33/16.5 kV, which was manufactured in 1976 (Tr2). Table 7 presents an expert diagnosis and EPI results for Tr2. As in the case of the analysis presented in Section 3.1, the diagnosis was based on the complete measurement data and visual inspection checklist issued after routine periodic tests. Additionally, in this case, the value of *k_i_* was set to 1.1 considering the age of Tr2. These data were used by the EPI method to perform the assessment.

Tr2 was detected with 12 types of faults: minor oil leaks, minor and major paint loss or corrosion, cooling system malfunction, desiccant fault, oil leaks from the cooling system, moisture in the oil, aged cellulose, aged oil, moisture in the solid insulation, winding asymmetry, and OLTS defects. Some of these faults are trivial and do not affect the exploitation perspective of the transformer, but some might be read as serious and potentially imply the possibility of serious consequences for the safe exploration of the transformer. In this case, EPI resulted in 108 (considering *k_i_* = 1.1). According to the proposed absolute EPI rating scale (Table 2), Tr2 has a rating of three and is classified as *Significant operational/investment measures required*. According to the relative rating scale, the EPI_%_ for Tr2 is 96_%_, which means that 96% of units in this population are in better technical condition than Tr2, and only 4% are in worse condition (Figure 5). According to HI1, Tr2 was assessed as in poor condition. In this case, a very poor condition was expected, as this unit had a number of serious defects. Unfortunately, the sensitivity of HI1 is deficient in this case, and some serious issues are masked. As a result, HI1 may provide an optimistic indication of the transformer’s condition. According to HI2, the score of Tr2 is 87, and it was the worst score in the analyzed population. On the other hand, EPI indicated that 4% of the population is in worse condition than Tr2. The economic aspect allowed the EPI to be more sensitive and accurate regarding this scenario.

## 4. Conclusions

This paper presents an alternative approach to the transformer assessment index. The EPI method was proposed and discussed. EPI is a relatively simple, dimensionless rating method for transformers. The proposed rating procedure is based on the results of actual periodical routine tests of the transformer, which makes it universal and easy to adopt for any transformer fleet. The method was designed for one of the European utilities where it has already been successfully implemented. As a result, its performance was verified in practice on 300 units of transformers. It was also compared with two different methods, and two representative use cases were described in this paper to confirm its usefulness. Regarding the presented study, some further conclusions may be drawn, as follows:

- EPI provides an absolute rating scale that corresponds with the current technical condition of the unit and its potential exploitation perspective;
- Simultaneously to the absolute rating scale EPI also provides a relative rating scale, which reflects the overall technical condition of the particular unit in the context of the entire fleet;
- Application of the EPI absolute rating scale requires gathering EPI for a representative sample of the population (ideally for all units in the population);
- EPI not only reflects the technical but also economic aspects of transformer maintenance;
- EPI can be potentially freely adopted for any transformer fleet, as well as for the specific situation of the utility, by adjusting the relevant parameters.

Apart from the advantages of the EPI mentioned above, some challenges and potential difficulties in application should be highlighted. Proposed *D*_i_ values are not universal and should be adjusted to the specific fleet in which this system is to be used, so implementation of EPI needs time, several optimization iterations, and expert knowledge. However, the weights proposed in this study can be used as a starting point or reference in any case. Furthermore, as EPI uses expert conclusions as input, it is also prone to any mistakes within the expert assessment. In the author’s opinion, one promising solution would be the integration of the EPI with one of the decision support expert systems designed for power transformers.

## Figures and Tables

**Table 1:** Description of the input parameters.

	Input Parameter	Definition
1	Minor oil leaks	Visual inspection indicated minor oil leaks in the transformer (other than 2, 11, 16, 17)—not relevant from the exploitation point of view
2	Major oil leaks	Visual inspection indicated major oil leaks relevant from the exploitation point of view (main tank, primary seal)
3	Minor paint loss or corrosion	Visual inspection indicated minor paint loss or corrosion, not relevant from the exploitation point of view
4	Major paint loss or corrosion	Visual inspection indicated major paint loss or corrosion, relevant from the exploitation point of view
5	Damage to the thermometer or invalid readings	Incorrect readings or damage to the upper oil layer thermometer were found
6	low oil level	Too low an oil level in the transformer conservator, below the permissible level
7	Damage to the oil level gauge or invalid readings	Incorrect, illegible readings, or damage to the oil level gauge
8	Buchholz relay fault	Damage or leaks or damage to the cables or lack of oil in the gas-flow relay (Buchholz)
9	Cooling system malfunction	Abnormalities in the operation of the cooling system (radiators, fans, control cabinet), other than 16
10	Grounding connection faults	Abnormalities in the connection and grounding of the transformer
11	Desiccant faults (dehydrating breather)	Abnormalities in the dehydrating system (leakage, moisture in the cartridge)
12	OLTC drive malfunction	Abnormalities in the operation of the PPZ drive
13	Bushing’s damage	Visual inspection indicated mechanical damage to the bushing (other than 17)
14	Signaling and controlling wiring faults	Damage to the transformer’s secondary and control circuits
15	Malfunction of the fiber optic temperature measurement system	Damage/abnormalities in the operation of the fiber-optic temperature measurement system of the active part of the transformer
16	Oil leaks (cooling system)	Oil leaks from radiators, pumps, valves or other components of the transformer cooling system
17	Oil leaks (bushings)	Oil leaks from bushings or their measuring taps
18	Moisture in the oil	Level of moisture in oil exceeded the allowed level
19	Aged oil	Aging markers of the oil indicate reaching the end of life or advanced aging process
20	Partial discharges	DGA results indicate PD
21	Overheating	DGA results and/or fiber optic temperature measurement results indicate overheating
22	Stray gassing	DGA results indicate stray gasses
23	Aged cellulose	Aging markers of the cellulose insulation indicate reaching the end of life or advanced aging process
24	Moisture in solid insulation	Level of moisture in solid insulation exceeded the allowed level
25	Aged bushings	Aging markers of the bushings indicate reaching the end of life or advanced aging process
26	Windings deformation	SFRA results indicate deformation of windings
27	Turn-to-turn short-circuits	Test results indicate turn-to-turn short-circuits
28	Windings asymmetry	Test results indicate winding asymmetry
29	Winding discontinuity	Test results indicate winding discontinuity
30	OLTC defects	OLTC time of non-simultaneous operation and/or head’s own time exceed the criteria values, and/or discontinuity on any tap detected
31	Magnetic circuit defect	Test results indicate a defect in the magnetic circuit

**Table 2:** Complete catalog of all possible input parameters for EPI and their proposed default values, *D_i_*.

*i*	Input Parameter AC	*D_i_*	*i*	Input Parameter DEF	*D_i_*
1	minor oil leaks (not within the main tank)	1	18	moisture in the oil	6.0
2	major oil leaks (main tank, primary seal)	2.0	19	aged oil	7.0
3	minor paint loss or corrosion	1.0	20	partial discharges (based on DGA)	7.0
4	major paint loss or corrosion	2.0	21	overheating	5.0
5	damage to the thermometer or invalid readings	0.1	22	stray gassing	0.2
6	low oil level	1.0	23	aged cellulose	60.0
7	damage to the oil level gauge or invalid readings	1.5	24	moisture in solid insulation	20.0
8	Buchholz relay fault	0.4	25	aged bushings	5.0
9	cooling system malfunction	2.0	26	windings deformation	25.0
10	grounding connection faults	0.5	27	turn-to-turn short-circuits	50.0
11	desiccant faults (dehydrating breather)	0.1	28	windings asymmetry	3.0
12	OLTC drive malfunction	2.5	29	winding discontinuity	6.0
13	bushing’s damage (visual)	3.0	30	OLTC defects	2.0
14	signaling and controlling wiring faults	2.5	31	magnetic circuit defect	38.0
15	malfunction of the fiber optic temperature measurement system	2.0			
16	oil leaks (cooling system)	1.0			
17	oil leaks (bushings)	1.0			

**Table 3:** Absolute EPI rating scale.

Rating	EPI	Group
1	<10	Does not require significant operational/investment procedures
2	10–50	Required maintenance/investments
3	>50	Significant operational/investment measures required

**Table 4:** Scoring matrix for the HI1 method.

Rating	HI1	Technical Condition
0	0–4	As new condition. Minimal Signs of ageing or deterioration
1	5–10	Good condition. Reliable operation expected for a lengthy period
2	11–16	Acceptable condition with significant signs of aging or deterioration. Consider condition-based maintenance
3	17–22	Poor Condition. Repair or replacement should be considered within the short term
4	23–28	Very Poor condition. High likelihood of failure.

**Table 5:** Scoring matrix for the HI2 method.

Rating	HI2	Technical Condition
0	0–27	Good
1	27–57	Average
2	57–100	Poor

**Table 6:** Expert diagnosis and EPI results for Tr1.

Expert Diagnosis	*i* (According to Table 1)	*D_i_* (According to Table 1)	EPI/EPI%	HI1	HI2
Moisture in the oil	18	6		12	34
Moisture in solid insulation	24	20	26/45%		

**Table 7:** Expert diagnosis and EPI results for Tr2 (*k_i_* = 1.1).

Expert Diagnosis	*i* (According to Table 1)	*D_i_* (According to Table 1)	EPI/EPI% ^1^	HI1	HI2
minor oil leaks (not within the main tank)	1	1	116/96% ^1^	21	87
minor paint loss or corrosion	3	1			
major paint loss or corrosion	4	2			
cooling system malfunction	9	2			
desiccant faults (dehydrating breather)	11	0.1			
oil leaks (cooling system)	16	1			
moisture in the oil	18	6			
aged oil	19	7			
aged cellulose	23	60			
moisture in solid insulation	24	20			
windings asymmetry	28	3			
OLTC defects	30	2			

^1^ considering *k_i_* = 1.1.

## Data Availability

Not applicable.
